# SSMap: A new UniProt-PDB mapping resource for the curation of structural-related information in the UniProt/Swiss-Prot Knowledgebase

**DOI:** 10.1186/1471-2105-9-391

**Published:** 2008-09-23

**Authors:** Fabrice PA David, Yum L Yip

**Affiliations:** 1 Swiss Institute of Bioinformatics, Swiss-Prot group, 1 rue Michel Servet, 1211 Geneva, Switzerland; 2 University of Geneva, Department of Structural Biology and Bioinformatics, 1 rue Michel Servet, 1211 Geneva, Switzerland

## Abstract

**Background:**

Sequences and structures provide valuable complementary information on protein features and functions. However, it is not always straightforward for users to gather information concurrently from the sequence and structure levels. The UniProt knowledgebase (UniProtKB) strives to help users on this undertaking by providing complete cross-references to Protein Data Bank (PDB) as well as coherent feature annotation using available structural information. In this study, SSMap – a new UniProt-PDB residue-residue level mapping – was generated. The primary objective of this mapping is not only to facilitate the two tasks mentioned above, but also to palliate a number of shortcomings of existent mappings. SSMap is the first isoform sequence-specific mapping resource and is up-to-date for UniProtKB annotation tasks. The method employed by SSMap differs from the other mapping resources in that it stresses on the correct reconstruction of the PDB sequence from structures, and on the correct attribution of a UniProtKB entry to each PDB chain by using a series of post-processing steps.

**Results:**

SSMap was compared to other existing mapping resources in terms of the correctness of the attribution of PDB chains to UniProtKB entries, and of the quality of the pairwise alignments supporting the residue-residue mapping. It was found that SSMap shared about 80% of the mappings with other mapping sources. New and alternative mappings proposed by SSMap were mostly good as assessed by manual verification of data subsets. As for local pairwise alignments, it was shown that major discrepancies (both in terms of alignment lengths and boundaries), when present, were often due to differences in methodologies used for the mappings.

**Conclusion:**

SSMap provides an independent, good quality UniProt-PDB mapping. The systematic comparison conducted in this study allows the further identification of general problems in UniProt-PDB mappings so that both the coverage and the quality of the mappings can be systematically improved for the benefit of the scientific community. SSMap mapping is currently used to provide PDB cross-references in UniProtKB.

## Background

The amino acid sequence constitutes the primary structure of a protein. While this primary structure can already provide useful hints on the function of a protein (e.g. conserved sequence motif), the complete understanding of a protein's function is hugely facilitated by the elucidation of its three-dimensional (3D) structure. Indeed, 3D structures have long proved to be useful for the understanding of important molecular recognition mechanisms such as enzyme catalysis [1], protein-protein interactions [2] or the role of mutations in human diseases [3]. Currently, information on a protein's primary sequence is comprehensively stored in the UniProt Knowledgebase (UniProtKB), which consists of the automatically annotated UniProtKB/TrEMBL section and the manually annotated UniProtKB/Swiss-Prot section [4]. 3D structures, on the other hand, are archived in the Protein Data Bank (PDB) [5]. To gather a comprehensive view of important protein features (including functional sites, post-translational modifications, and mutations), users can either consult the "Sequence annotation (Features)" section of a UniProtKB entry, or look up the 3D atomic section of the PDB file. Except for users familiar with 3D structures, it is clearly not a straightforward procedure to retrieve important information from structural coordinate files. A variety of tools and resources already exist to help users to interpret 3D structures [6-8]. However, most – if not all – of these tools provide information relative to a residue number on a protein chain, which unfortunately, does not always match the amino acid number in the corresponding protein sequence in UniProtKB. As a consequence, users are currently facing two challenges to gather useful information on a protein both from the sequence and structure levels. First, they have to identify the exact protein structure which corresponds to their protein of interest; and second, they have to know the correspondence between residues on a protein chain in the PDB file and those on the UniProtKB primary sequence. Indeed, it is not at all a trivial task to provide an accurate UniProt-PDB mapping down to the residue level.

In UniProtKB/Swiss-Prot, the usage of 3D-structures is an important complement to the literature for the high quality annotation of proteins [4,9]. The database also strives to help users to overcome the challenges mentioned above. Therefore, manual annotation of protein structures in UniProtKB/Swiss-Prot involves first, ensuring a complete and accurate coverage of cross-references to these structures in the PDB and second, exploiting the structural data to annotate protein features. Cross-references to PDB are indicated in the cross-reference section (DR PDB line in the flat file of UniProtKB). Each DR PDB line references one taxonomically matched structure available from PDB. The line indicates the PDB entry code, the name of the PDB chain(s) matched to the reference sequence (sequence shown in the UniProtKB entry), the experimental method used, the resolution (when available) and the boundaries of these matches on the UniProtKB reference sequence. Both the correct association of a PDB chain to a taxonomically-matched UniProtKB entry and the alignment are thus essential for cross-referencing. A precise alignment is also important for residue-residue level mapping which is crucial for the correct annotation of protein features and functions using structural information.

Since 2001, the Macromolecular Structure Database (MSD) has been producing a mapping in collaboration with UniProtKB through the SIFTS (Structure integration with function, taxonomy and sequence) initiative [10]. The mapping is obtained via a rather complex procedure which consists of assembling alignments of resolved segments into full-length alignments . This mapping is used to automatically produce the PDB cross-references (DR PDB lines) in UniProtKB. Throughout the years, the quality of SIFTS mappings has been improved. However, manual curation is still constantly required to validate or correct these cross-references by adjusting the boundaries of PDB matches on UniProtKB sequence and/or re-attributing a PDB cross-reference to another UniProtKB entry. Feedback on such modifications is given to the MSD. However, with the increasing number of experimental structures resolved, this curation process is becoming more and more difficult to follow. It is also difficult to pinpoint exactly the source of these errors so that improvement can be made in the SIFT mapping process. One possible solution to this problem would be the use of an independent mapping resource to identify and compare the differences.

Several other mapping resources exist. In 2005, Martin published PDBSWS [11], a new mapping between UniProtKB and PDB, which aimed to be specially fast and up-to-date. The method uses cross-references both in UniProtKB and in PDB to attribute PDB structures to UniProtKB entries. As a result, this mapping is not completely independent from the mapping provided by MSD and thus cannot be used to aid in the curation or verify the quality of cross-references in UniProtKB. In the same year, a web resource Seq2Struct [12] was described to provide links between UniProtKB sequences and PDB structures. This resource, however, does not provide residue-residue mapping and is not updated since September 2006. More recently, MMDB introduced a new tool to help users to easily visualize annotated features in RefSeq sequences on 3D-structures [13]. This visualization tool is clearly based on a residue-residue level sequence to structure mapping. The mapping is however not directly available for download in a large-scale manner.

In this study, a new UniProt-PDB mapping, SSMap (Sequence – Structure Mapping) was generated. The primary objective of this mapping is to improve the quality of structure-related information in UniProtKB: both at the level of cross-references to PDB and subsequent feature annotation. In order to achieve this, it is worth highlighting several characteristics of SSMap. First, the mapping method used in SSMap diverges from those employed in SIFTS and PDBSWS. The method concentrates on the quality of the reconstruction of the PDB sequence from structures and on the correct attribution of a UniProt entry to each PDB chain by using a series of post-processing steps. Because of this difference in methodology, an objective and systematic comparison with other mapping data can be conducted. Second, all the alignments made between a UniProtKB sequence and PDB structures susceptible to be mapped (sharing over 70% sequence identity) are kept in SSMap for quality verification and curation purposes. Third, mappings for alternative sequence forms (e.g. splice isoforms and inteins) are provided to facilitate isoform-specific annotations. Finally, the mapping procedure is automated and kept up-to-date with each UniProt release so that accurate information is provided to the users even with the constant increase in sequence and structural data.

In the following text, we present in more detail a comparison between mappings provided by SSMap and the other available resources (SIFTS, PDBSWS and DR PDB). The results of the comparison should be able to identify systematically common and different mappings. Common mappings could be used as input for (semi)-automatic annotation processes, as mappings confirmed by several methods have a higher confidence. Different mappings, on the other hand, allow to identify the problems inherent in each mapping and consequently, improve the quality of each of them.

## Methods

### Reconstruction of sequences from PDB structures

PDB structures are composed of one or several macromolecular chains. From each chain of amino acids, a one-letter code sequence is derived. The sequence reconstruction program was written to specifically deal with the existence of gaps in PDB structures (i.e. unresolved regions) as well as to cover and palliate a maximum number of exceptions in PDB files. The process consisted in several steps. In the first step, draft sequences were reconstructed from ATOM lines. In the event of modified residues, PDB annotation available in MODRES lines was used to determine the original amino acid type. Gaps in sequences were deduced from residue numbering gaps in the structure, backbone N-C inter-atomic distances (or Cα-Cα distance if not available) and covalent bond angles. For the two latter criteria, the canonical values found in the literature [14] – with a degree of tolerance – were used. In the second step, a pairwise alignment was done between the reconstructed sequence and the sequence extracted from PDB SEQRES records. The SEQRES sequence corresponds to the sequence of the protein that was used during the experiment. Gaps in this alignment were adjusted to better fit with unresolved regions deduced in the first step. Finally, gaps in the reconstructed sequence were filled using the SEQRES sequence. This transfer of information (from SEQRES to reconstructed sequence) helped add in the final sequence the possible unresolved regions as well as the N- and C-terminal unresolved extents. Compared to simply taking the sequence from the SEQRES record, our procedure offered two main advantages. First, the resolved/unresolved status of each residue was recorded during the process; and second, information was obtained primarily from ATOM lines which are in general more reliable than the SEQRES records. It is expected that this reconstruction procedure will yield the most probable sequences of the proteins used in the experiment.

### Mapping

**Figure 1:**
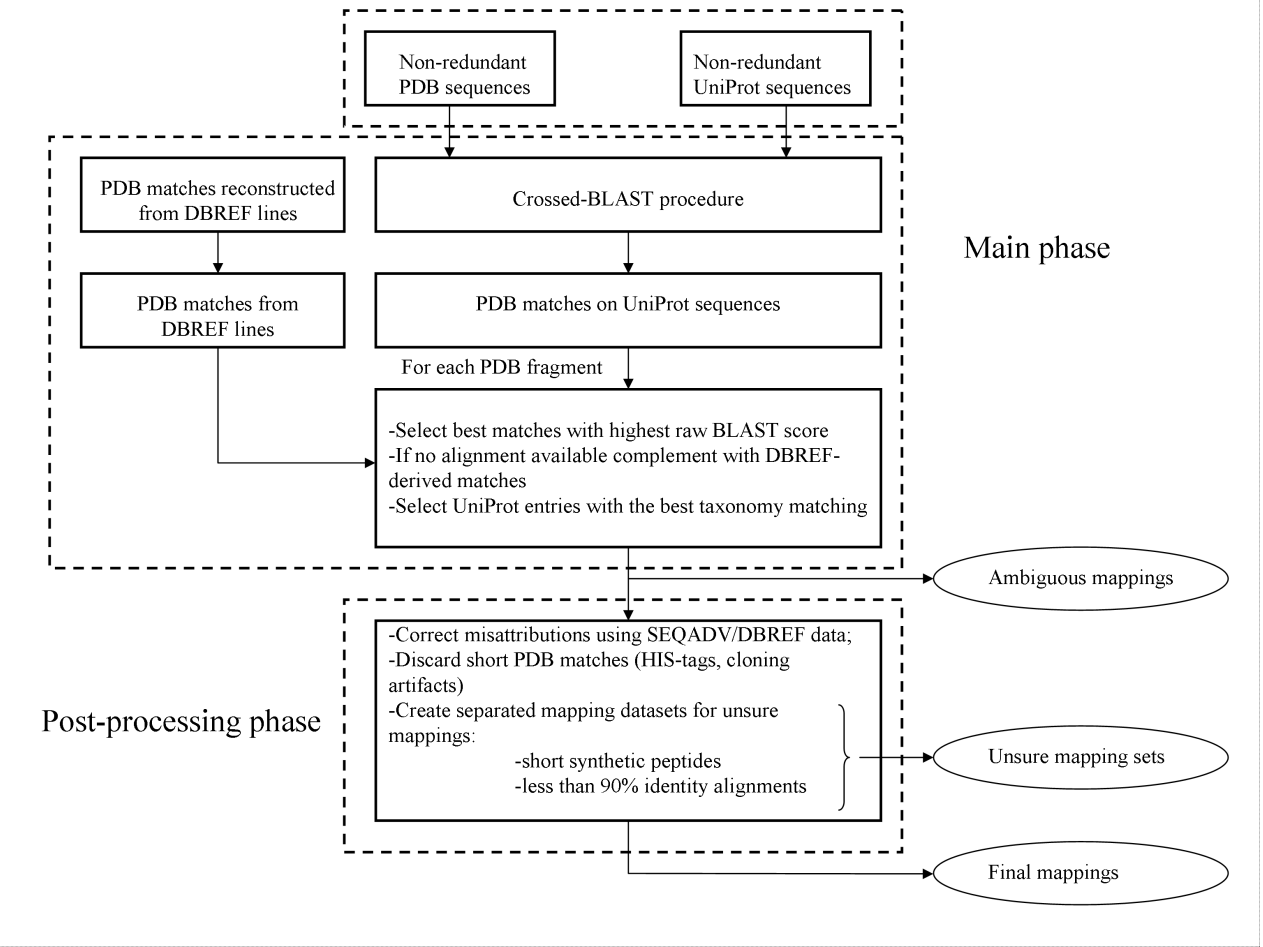
Flow chart of the SSMap mapping.

The mapping procedure was outlined in Figure 1. The main product of this procedure is a non-ambiguous residue-residue mapping between UniProtKB sequences (including alternative sequence forms) and PDB structures sharing at least 90% sequence identity.

#### Searching PDB reconstructed sequences similar to each UniProtKB sequence

The aim of this step is to get all the matches between non-redundant PDB reconstructed sequences and non-redundant UniProtKB sequences sharing at least 70% sequence identity. Non-redundancy of PDB chains was defined based on both the amino acid sequence and the unresolved/resolved status of each amino acid. We performed, successively, BLAST searches of the reconstructed PDB sequences against UniProtKB sequences and then of the UniProtKB sequences against the reconstructed PDB sequences. This cross-BLAST procedure was necessary to ensure the finding of best matches and to maximize mapping coverage.

In parallel, cross-references to UniProtKB in PDB records (the field DBREF) were used to complement the BLAST search result in order to obtain remote (less than 70% sequence identity) or short matches.

#### Attribution of PDB chains to UniProtKB sequences

**Figure 2:**
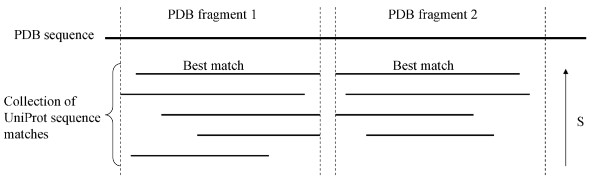
**Definition of PDB fragments and best matches from BLAST hits**. Matches are ordered by raw BLAST score (S) in order to select best matches for each PDB fragment.

The attribution procedure was specific to UniProtKB sequences (including alternative sequence forms) and was composed of four phases. First, PDB reconstructed sequences were divided into fragments when different UniProtKB sequences matched on distinct (non-overlapping) parts of the reconstructed sequences (Figure 2). This division of sequences into fragments allowed chimeric proteins to be detected for example. Second, for each fragment of PDB reconstructed sequences, best match(es) (i.e. match(es) which have the highest BLAST raw score (S)) on UniProtKB sequences were identified. Third, for each pair (UniProtKB sequence, PDB chain) flagged as best match, taxonomy data were retrieved from the UniProtKB and PDB entries. Only the set of best matches with the best taxonomy matching between the corresponding UniProtKB and the PDB entries was selected. The best taxonomy matching corresponds to the closest distance in the taxonomic tree between the taxons described in an UniProt entry and the PDB entry. Finally, among this final set of matches, if only one UniProtKB/Swiss-Prot entry was represented, then the non-ambiguous attribution was made to it. If there were several UniProtKB/Swiss-Prot entries, or several UniProtKB/TrEMBL and no UniProtKB/Swiss-Prot entries, then no automatic attribution was performed and the information was stored separately. When there were no alignments available for a given PDB chain, the attribution was done using the computed DBREF matches if there was only one matching UniProtKB entry. This step was especially useful for variable segments of immunoglobulins, MHC proteins, viral proteins and small peptides.

At the end of this procedure, if two UniProtKB alternative sequence forms of the same UniProtKB entry match equally well to a PDB chain, both mappings were considered valid.

#### Post-processing of the mapping

Post-processing of mappings aimed to correct special cases for which the attribution method, which relied mainly on the raw BLAST score, failed to find the correct mapping. The process also identified mappings that were not relevant for annotation. Several points were taken into consideration.

First, attributions could be incorrect when a directed mutagenesis resulted in a sequence which mimicked an orthologuous sequence. Therefore, in cases where the taxonomy was not the same between PDB and UniProtKB/Swiss-Prot, we proceeded by searching to see if there was an alignment of lower score in the set of BLAST alignments or the set of DBREF matches that satisfied better the taxonomy match. For these new potential matches, the number of engineered mutations reported in SEQADV records of the PDB file header was used to evaluate their relevance.

Second, PDB matches of less than 100 residues containing poly-histidines (at least 3 consecutives H or HQ repeats) were filtered out because they corresponded to cloning artifacts. For the same reason, PDB matches shorter than 30 residues were discarded unless another fragment of the same PDB chain mapped to a UniProtKB entry.

Two separate mapping datasets were also created to store cases of PDB matches suspected to be uninformative or uncertain. These datasets were planned to be checked manually later. The first dataset consisted of PDB matches involving short synthetic PDB sequences with an imperfect taxonomy correspondence. The second dataset consisted of PDB matches involving alignments with less than 90% sequence identity.

The final mapping obtained after post-processing was supported by alignment with at least 90% sequence identity.

### Storing data

All the data necessary for the mapping and its evaluation were stored in a PostgreSQL relational database consisting of 3 main schemata.

In the first schema, PDB reconstructed sequences and derived data (e.g. residue numbering in the structure, modified residue status, unresolved residue status) were stored with taxonomic data, annotated features and experimental conditions parsed from the PDB files. Basic UniProtKB data (e.g. sequence, entry accession code, taxonomy identifier) were loaded in a second schema. Finally, in a third schema, BLAST alignments were stored in a compressed form and indexed by reconstructed PDB sequence unique identifiers and UniProtKB sequence unique identifiers. Final mappings, unsure and ambiguous mappings were stored explicitly in other tables.

### Incremental updates and update frequency

New UniProtKB sequences and new PDB reconstructed sequences are added to the database during incremental update. Equivalent cross-BLAST procedures, as described above, are then performed only for the new UniProtKB and PDB reconstructed sequences. The attribution of PDB chains to UniProtKB entries (and alternative sequence forms) is rerun each time. Incremental updates take two days and are performed every three weeks.

### Evaluation of SSMap

The version of SSMap used for the comparison was built using the public versions of UniProtKB (release 11.2) and PDB (28^th ^June 2007). This version of UniProtKB was composed of 272,212 (6%) UniProtKB/Swiss-Prot entries and 4,464,302 (94%) UniProtKB/TrEMBL entries. PDB contained 102,203 protein chains in 43,912 entries.

The mapping was evaluated by comparing with several other mapping data:

1- PDBSWS, the mapping provided by Martin's group [11] (version of mid-June 2007). This mapping is released in a rigid-column format file.

2- The mapping provided by the MSD through the SIFTS initiative [10] (version of 18^th ^June 2007). This mapping is released in the form of XML files, where each file corresponds to one PDB entry.

3- The DR PDB lines in UniProtKB (UniProtKB release 11.2).

We note that although MSD provides UniProtKB DR PDB lines, DR PDB lines benefit continually from manual verification and correction. The update frequency of DR PDB is not the same as SIFTS neither. Consequently, SIFTS and DR PDB constitute two different datasets for comparison.

As a starting point, the raw data obtained from PDBSWS and SIFTS (residue to residue mapping) were first processed to generate alignments comparable to those in SSMap. PDBSWS residue-residue mapping did not take into account unresolved amino acids in the PDB file. Consequently, internal gaps (when mapped to UniProtKB sequence) were filled with Xs when reconstructing the alignment. For data from SIFTS, it was found that some PDB chains were split into several segments. We assumed these to be the equivalent of our PDB fragments and thus, distinct alignments were reconstructed for each fragment from SIFTS. For both SIFTS and PDBSWS, 3-letter code residues, which did not correspond to standard residues, were replaced by 'X' in the corresponding 1-letter code sequence.

For comparison, the number of common mappings (i.e. same (UniProtKB entry, PDB chain) pairs) between SSMap and the other sources was computed. Mappings present only in SSMap were considered either as new or alternative mappings with respect to the resource they were being compared with. 'Alternative' means that the PDB chain was mapped to another UniProtKB entry. Missing mappings in SSMap were also identified. For common mappings between SSMap and the other sources of mapping, equivalent local alignments (i.e. with the same (PDB chain, UniProtKB entry) pair) were further compared to check for their quality. Alignments were considered identical if there were no differences in gap position and if the sequences were exactly the same. For equivalent but non-identical alignments, both alignment length and boundaries (positions of C- and N-terminal residues on UniProtKB sequence) were compared.

## Results

### SSMap statistics

Sequences of the 102,203 protein chains in 43,912 PDB entries were reconstructed. This corresponded to 49,942 non-redundant PDB reconstructed sequences that were used as input for the BLAST procedure. From the resulting alignments, 93,003 mappings (non-redundant (UniProtKB accession, PDB entry chain) pairs) were computed. The majority (84%) of them were mappings of PDB chains to UniProtKB/Swiss-Prot entries. The rest (16%) were mappings to UniProtKB/TrEMBL entries. This reflected the effort of Swiss-Prot curators to preferentially annotate proteins with resolved 3D structures. Among these mappings, 486 (0.7% of UniProtKB/Swiss-Prot mappings) were specific to splice isoform sequence(s), and 17 were specific to intein processed sequences.

From the UniProtKB perspective, 4% (11,239) of all UniProtKB/Swiss-Prot entries were matched to at least one PDB structure with 100% identity (taxonomy not considered). If one considered all alignments with more than 90% and 70% sequence identity, this percentage increased to 19% (52,397) and 28% (78,259) of all UniProtKB/Swiss-Prot entries, respectively. This highlighted the potential wide impact of structural information on UniProtKB/Swiss-Prot entries. In the final mapping (taxonomic-specific), 14'987 UniProtKB/Swiss-Prot entries are represented.

**Figure 3:**
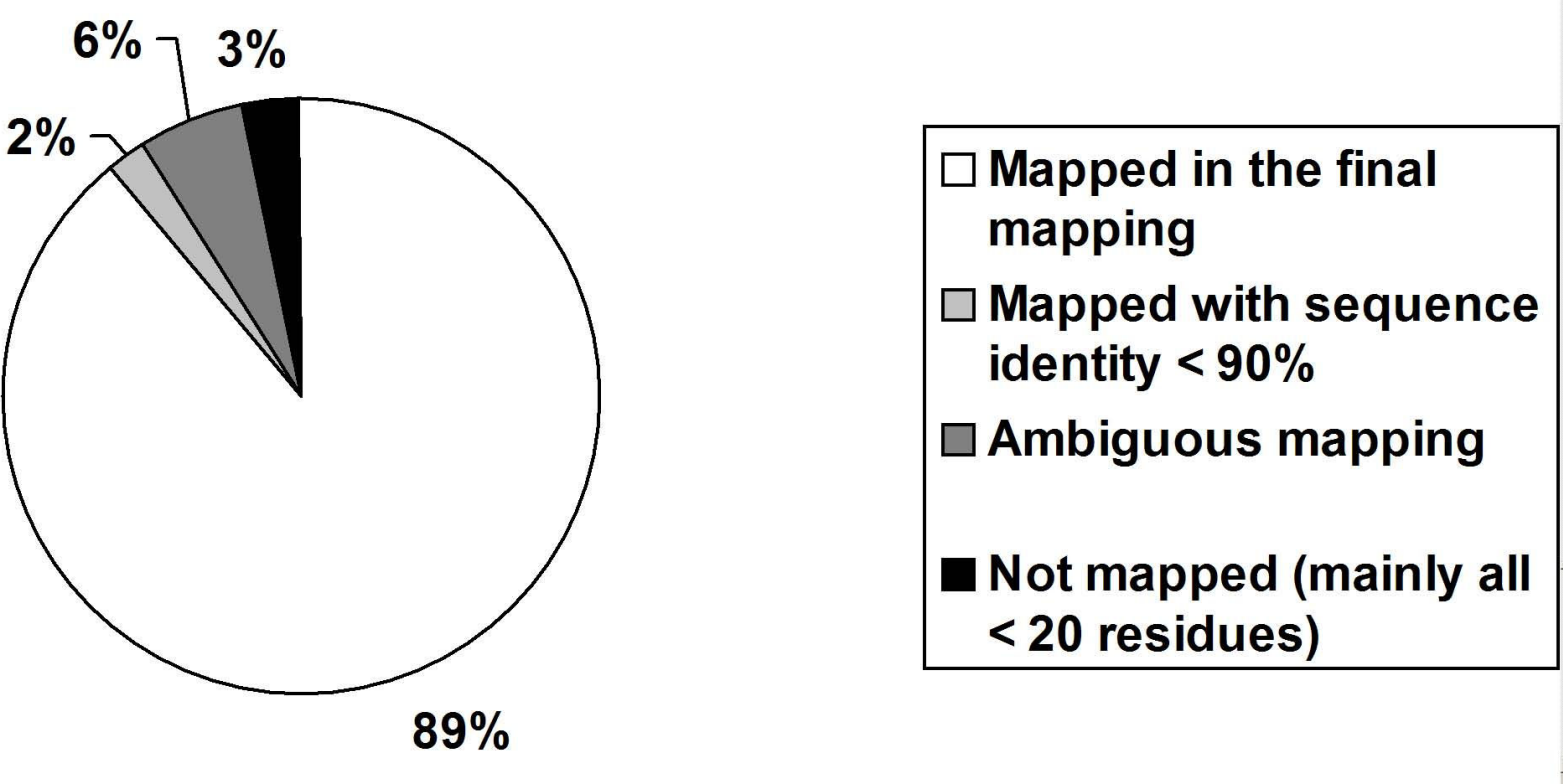
**Pie chart representing the proportion of protein PDB chains mapped and unmapped in SSMap**.

From the PDB perspective, 89% of all the protein PDB chains (90,923) were mapped unambiguously to at least one UniProtKB entry with at least 90% sequence identity (Figure 3). Among these 90,923 mapped PDB chains, 126 were mapped unambiguously to several UniProtKB entries. In all these cases, different fragments of PDB chains were mapped to a different UniProtKB entry. These corresponded mostly to immune system or viral proteins with a high conservation (sequence identity > 90%), or to fusion proteins (e.g. PDB:1R6Z chain Z or PDB:2JAD chain A). About 2% (1,741) of the PDB chains were only supported by alignments with a sequence identity lower than 90%. There was ambiguity for 6% (6,792) of the PDB chains, where possible attribution to several UniProtKB entries existed (Figure 3). A small number (61) of PDB chains were small synthetic peptides not mapped at the taxonomy level to UniProtKB entries. For these 3 last PDB chain datasets, associated mappings could not be validated automatically and were thus not included in the final mapping results. The remaining 3% (2,918) of protein PDB chains were not found at all among the available SSMap alignments (sequence identity greater than 70%). Among these ones, nearly all (2,628) chains were shorter than 20 residues; the rest (290) often contained modified/unknown residues or presented unresolved segments.

### Comparison of mappings

In the following section, we compared the number of mapped PDB chains and the pairwise attribution of PDB chains to UniProtKB entries between SSMap and the other mapping resources. As mappings specific to splice isoform and inteinized sequences were not present in SIFTS, PDBSWS and DR PDB, the comparison was only made at the level of UniProtKB entries.

PDBSWS provided the highest number of mappings (Table 1), both in terms of PDB chains mapped and the mapping pairs. This was followed by SSMap, SIFTS and DR PDB. In SIFTS, the number of mappings ((UniProtKB entry, PDB chain) pairs) was equal to the number of PDB mapped chains (Table 1), indicating that multiple mappings of a PDB chain on different proteins (chimeric proteins or immune system proteins) were not supported in SIFTS. This was in conflict with the literature [6], which reported that SIFTS dealt with such cases. To verify this observation, we reviewed the 126 cases in SSMap where the PDB chains were mapped unambiguously to several UniProtKB entries. We found that in 85 cases, the corresponding XML files did not exist in SIFTS. For the remaining 41 cases, the PDB chains were only mapped to one UniProtKB entry in SIFTS.

**Table 1:** Statistics for the different sources of mappings.

Data type\Mapping source	SSMap	DR PDB	SIFTS	PDBSWS
1. Number of mappings (AC, PDB chain)	91,050	78,238	84,398	93,167
2. Number of PDB chains mapped	90,923	77,711	84,398	93,141

Mappings correspond to non-redundant pairs of UniProtKB accession number (AC) and PDB chain.

Before trying to understand the reasons behind the differences in mappings, we checked if all the mappable PDB chains were present in SSMap. For this, we simply verified if the mapped PDB chains in other resources existed in SSMap's PDB schema. It was found that there were 35, 412 and 205 chains present in DR PDB, SIFTS, and PDBSWS, respectively, but absent in SSMap. All these chains present in DR PDB, SIFTS and PDBSWS were found to be either obsolete or unreleased PDB chains, and thus would probably disappear from these resources in their next update.

In the following result sections, we will try to document the reasons behind the differences in mappings.

### Comparison of PDB chain attributions in the different mapping sources

We compared the attribution of a UniProtKB entry to a PDB chain, or (UniProtKB AC, PDB chain) pairs, between SSMap and other resources. On average, SSMap had about 80% of common mappings with other resources. The common mapping set was largest between SSMap and PDBSWS (85% of all SSMap mappings) (Table 2). This could be simply due to the fact that there were more mappings in PDBSWS than in SIFTS or DR PDB (Table 1). In fact, we noted at the same time that there were many more new mappings found by SSMap when compared to SIFTS or DR PDB (Table 2).

**Table 2:** Comparison between different sources of mappings.

Reference source	SSMap		
Compared source	SIFTS	PDBSWS	DR PDB

1. Common	72,543 (80%)	78,178 (85%)	68,692 (78%)
2. Alternative	5,548 (6%)	7,736 (8%)	4,155 (5%)
3. New	12,959 (14%)	5,143 (7%)	18,472 (17%)
4. Missing	5,931 [5,733]	7,147 [7,003]	5,362 [5,206]

SSMap was taken as the reference dataset. The number indicated the number of mappings and the percentage was calculated based on the total number of mappings (91,050) in SSMap. 1) Common: mappings (AC, PDB chain) common in both datasets being compared. 2) Alternative: mappings in which a PDB chain was mapped to different UniProtKB entries in the compared sources. 3) New: mappings in SSMap. The PDB chain was not mapped in the compared database. 4) Missing: mappings that were missing in SSMap. The total number of mappings that were found as ambiguous/unsure mappings in SSMap was indicated between brackets.

NB: The sum of common, alternative and new mappings was not exactly equal to the total number of mappings in SSMap because of the fusion protein cases. Some mappings were counted twice in different categories.

Between 5–8% of the SSMap mappings were alternative mappings when compared to other sources. In order to better evaluate the reasons for these alternative attributions, we manually verified random sets of alternative mappings taken from each pairs of resources that were being compared (100 SSMap vs SIFTS, 50 SSMap vs PDBSWS and 50 SSMap vs DR PDB). Table 3 shows the result of this evaluation. Overall, about 60% alternative mappings corresponded to good attributions of SSMap in comparison to other resources. For example, PDB:1QI0 chain A (Endoglucanase cel5A from Bacillus agaradhaerens) is mapped to UniProtKB:P06565 (Endoglucanase B from Bacillus sp. (strain N-4/JCM 9156)) in SIFT, but to UniProt:O85465 (Endoglucanase 5A from Bacillus agaradhaerens) in SSMap. About 20% alternative mappings were ambiguous in that it was not possible to define which mapping was the best only from the data in the PDB files. Often, contradictory information in different fields of the PDB file prevented a sure attribution and it was necessary to read the associated scientific article. The last 20% alternative mappings were errors in SSMap. These corresponded mainly to wrong attributions in limit cases. For example, the result of BLAST alignment sometimes contained one or several mismatched residues at the N-terminal. This increased artificially the length of the alignment and thus the score computed by SSMap. It was also noted in cases where SIFTS proposed erroneous alternative mappings when compared to SSMap, half of these involved obsolete UniProtKB entries. While it is not possible to draw definite conclusions regarding the quality of each mapping resource with these small data subsets, our results did suggest that SSMap might propose a better solution in cases where the different resources disagreed.

**Table 3:** Results of evaluation for alternative mappings.

Reference source	SSMap		
Compared source	SIFTS	PDBSWS	DR PDB
Correct in SSMap	67 (67%)	29 (58%)	33 (66%)
Error in SSMap	17 (17%)	8 (16%)	10 (20%)
Ambiguous	16 (16%)	13 (26%)	7 (14%)

Random chosen sets of alternative mappings from each comparison pairs (100 SSMap vs SIFTS, 50 SSMap vs PDBSWS, 50 SSMaps vs DR PDB) were evaluated manually.

With regards to new mappings, the number of new mappings in SSMap – as compared to DR PDB – was the largest (18,472). These 18,472 mappings corresponded to 7,793 PDB entries. As DR PDB has not been updated as recently as SSMap, we analyzed how recent these 7,793 PDB entries were. Surprisingly, it was found that a non-negligible number of the structures (2,863) were published before 2006 and thus should normally be present in DR PDB. As an example, for 1,226 of the PDB structures released in 2005, the corresponding DR PDB lines were still missing in UniProtKB. The reason for this is unclear. This indicated that the new mappings provided by SSMap were not merely the result of a more recent update of SSMap. A random set of 200 new mappings was manually analyzed. Almost all (198) were good mappings. The remaining two were due to the fact that the corresponding protein sequences were never deposited in any sequence database (including UniProtKB) and the mapping process mapped the PDB chain to its nearest taxonomic neighbor (e.g. PDB:1ZOY chain A (FAD-binding protein from pig) was mapped to UniProtKB: Q2HJI1 (SDHA protein from cow), and PDB: 1ZOY chain C (large cytochrome binding protein from pig) was mapped to UniProtKB: Q99643 (Succinate dehydrogenase cytochrome b560 subunit, mitochondrial, from human)).

SSMap was found to have about 6,000 missing mappings when compared with all the other sources. Of these missing mappings, over 90% were deliberately excluded from SSMap because attribution was ambiguous or uncertain (Table 2). The remaining missing mappings in SSMap were analyzed in more detail. When compared to DR PDB, it was found that 128 out of the 156 missing mappings were sequences shorter than 20 residues. For these cases, the crossed-BLAST procedure failed. There were 18 mappings in DR PDB that involved obsolete PDB chains (e.g. PDB:2PWN chain B) no longer present in PDB, and 17 mappings with non-amino acid chains or chains with no atomic coordinates (e.g. PDB:1D8SF or PDB:1J01J). Finally, the rest (11) were mappings with a percentage of identity lower than 70%. Similar result was noted when compared to PDBSWS. In particular, 125 out of the 144 missing mappings corresponded to PDB sequences shorter than 20 residues, and 10 were mappings with a low sequence identity (<70%).

### Comparison of local alignments supporting the mapping

The comparison of alignments was done on common mappings between SSMap and the other sources of mapping.

According to Table 4, the majority of the SSMap alignments differed from those in SIFTS (68%) or PDBSWS (78%). Most of the time, this was due to the fact that the reconstructed sequences from PDB were not exactly the same in each resource. For example, it was observed that among the 49,199 non-identical alignments in SIFTS, 92% (45,371) were due to the presence of unresolved or modified residues noted as 'X' in the SIFTS reconstructed sequences. To be independent of such sequence variations, we proceeded by checking the length differences of those non-identical alignments (Table 5). It was found that the number of alignments presenting a different length was much higher between SSMap and PDBSWS (81%) than between SSMap and SIFTS (17%). This was due to the fact that PDB reconstructed sequences in PDBSWS did not contain unresolved N- and C-terminal regions as in SIFTS or SSMap. Overall, the alignments between SSMap and SIFTS were of comparable length. Alignments with a length difference of more than 5 residues represented only 2% of the total non-identical alignments. Among these alignment pairs, 2/3 of the cases correspond to a longer alignment in SSMap.

**Table 4:** Evaluation of equivalent alignments pairs between SSMap and the two resources SIFTS and PDBSWS.

	SIFTS	PDBSWS
Number of equivalent alignments	72,158	77,818
Number of identical alignments	22,959 (32%)	16,775 (22%)
Number of non-identical alignments	49,199 (68%)	61,043 (78%)

Equivalent alignments corresponded to the same (UniProtKB entry, PDB chain) pairs in both mapping sources. Mappings involving isoform sequences were not taking into account.

**Table 5:** Evaluation of non-identical alignments between SSMap and the three other resources.

	SIFTS	PDBSWS	DR PDB
Total number of non-identical alignments	49,199	61,043	(68,692)*
**Comparison of length**			
Different lengths	8,463 [17%]	49,364 [81%]	NA
| Length difference| <= 5	7,004 (83%)	25,050 (51%)	NA
| Length difference| > 5	1,459 (17%)	24,314 (49%)	NA
**Comparison of boundaries**			
Different boundaries	16,824 [34%]	50,362 [82%]	10,848
| Boundaries diff Nterm| <= 5	15,666	34,302	10,058
| Boundaries diff Cterm| <= 5	16,146	36,163	10,324
Boundaries diff Nterm < -5	473	978	363
Boundaries diff Nterm > 5	674	13,317	445
Boundaries diff Cterm < -5	431	11,752	273
Boundaries diff Cterm > 5	221	621	219

The difference in length was calculated by subtracting the alignment length of the compared resource from that of SSMap. Boundaries differences were computed similarly. The percentages between square brackets were computed relatively to the total number of non-identical alignments. The percentages between round brackets were computed relatively to the number of alignment pairs having a different length or different boundaries.

NB: *Since DR PDB did not provide alignment, the number indicated as reference was the total number of common (equivalent) mappings between SSMap and DR PDB.

Apart from alignment lengths, the differences in boundaries between alignments were also evaluated. Boundaries represented the UniProtKB residue number onto which the end points of the PDB reconstructed sequence fragment mapped. Although DR PDB lines did not provide full alignments for comparison, boundaries were indicated in these lines and were included in this comparison (Table 5). It was found that among the common mappings between SSMap and DR PDB, 2,947 matches (or 1,907 DR PDB lines) contained no information on boundaries. For the remaining mappings, 10,848 (16%) presented different boundaries, and only in 1,084 of them the variation was greater than 5 residues. We analyzed manually a set of 50 of these extreme cases. In more than half of the cases, the regions covered by SSMap boundaries were longer. Often, these corresponded to better-reconstructed sequences in SSMap that allowed the boundaries to be correctly located beyond what was indicated in DR PDB (e.g. PDB: 2BYU chain H was mapped to UniProtKB: Q41560 residues 42–151 in SSMap instead of residues 43–137 in DR PDB). In other cases, this was due to the fact that artifact sequences (cloning artifacts, His-tags) in the N- or C-terminal of the reconstructed sequences were aligned by chance to a UniProtKB sequence. These resulted in incorrect boundaries assigned by SSMap. In cases where the regions covered by SSMap boundaries were shorter, they corresponded often to a split of the alignment into 2 distinct ones in SSMap. This happened when, for instance, a linker had been artificially inserted into the sequence to link 2 non-consecutive segments of the same protein. For example, in the PDB entry 1MQD chain D, two segments of the UniProtKB protein P19491 were artificially linked together. While SSMap mapped this PDB entry to residues 413–527 and 653–794 on P19491, DR PDB mapped these to 413–794. To compare the boundary differences between SSMap and SIFTS, the same kind of analysis was carried out. A new set of 50 cases was analyzed. It was found that in 34 out of these 50 cases, the boundaries indicated by SIFTS were wrong. SSMap was wrong in 9 cases and for the remaining 7, boundaries indicated by both resources appeared to be acceptable. Among the 34 cases for which SIFTS was wrong, we found that 18 cases were due to the presence of discontinous parts in the sequences. This could either be related to the presence of a long linker as explained above, or in some rare cases, an inversion of the sequence after post-translational modification. For example, the two parts of the concanavalin A (UniProtKB entry P02866) were inversed due to post-translational modification. SSMap correctly mapped the PDB entry 1JUI chain D residues 119–237 and 1–118 to P02866 residues 30–148 and 164–281, respectively. In SIFTS, 1JUI chain D residues 1–127 were mapped to P02866 residues 164–290, the mapping for the rest part of the chain D was missing. Finally, for the PDBSWS dataset, the difference in boundaries could be once again largely explained by the absence of unresolved N- and C-terminal regions in PDBSWS.

## Discussion

Sequence to structure mapping is an essential component for any protein feature analysis in a structural context, and for protein structure analysis or structure modeling using sequence information. In this study, SSMap, a new UniProt-PDB mapping, was built in order to improve the quality of structure-related information in UniProtKB – both at the level of curating cross-references to PDB and subsequent feature annotation. The mapping strategy used in SSMap is different from those used by other resources. As such, SSMap is an independent mapping resource which further allows one to identify and pinpoint current weaknesses or strengths of existent mapping resources.

It was noted that two main steps in the mapping procedures – sequence reconstruction and alignment method – have a profound influence on the final mapping results. The main difference between SIFTS and SSMap appears to reside in the order of execution of these steps as well as on the emphasis put on them. In SSMap, we believe that the reconstruction of sequences from structures, especially the treatment of unresolved regions, is important for good quality alignments and subsequent mapping. Therefore, unresolved residues in PDB files were first deduced from the SEQRES or MODRES records, as well as from information in ATOM lines, so that the most complete reconstructed protein sequence reflecting the one used in the experiment could be obtained. This complete sequence was used subsequently in a standard BLAST search against UniProtKB (and vice versa) to ensure that the best alignments were found for the subsequent mapping steps. Conversely, in SIFTS, alignments were performed on resolved segments first. These resolved segments were first aligned against either the sequence from the SEQRES records or the UniProtKB sequence. The respective separate alignments for these segments were then merged to assemble the full-length alignments. The complete residue-level mapping between the sequence of the complete protein from the experiment and its UniProtKB counterpart was only obtained by merging the two composite alignments (one with SEQRES, one with UniProt) . Thus, the SIFTS procedure was relatively complex as multiple separate alignments were assembled and merged. These differences in the methods were reflected in our results. In particular, when we analyzed manually the cases in which the difference in boundaries between SIFTS and SSMap exceeded 5 residues, it was found that where SIFTS was incorrect, a non-negligible number was due to the fact that PDB chains contained discontinuous sequence segments.

The mapping procedure in PDBSWS was less comparable to SSMap as the former partly relied on existing cross-references from PDB (DBREF) and UniProtKB (DR PDB). However, it should be noted that while SSMap also used the DBREF information to post-process the mapping when there was a taxonomy disagreement or when the chains to be mapped were too short, extra care was taken to realign the PDB chain to the referenced UniProtKB sequence and to verify that the resultant alignments were better than those in the initial mapping. Wrong or ambiguous taxonomy information indicated in the PDB file was thus identified. Although PDBSWS's mapping procedure is relatively simple, good quality mappings are generally provided. The mapping, however, lacks the N- and C-terminal unresolved regions, which are both essential to define the correct boundaries in DR PDB.

The quality of the mappings in SSMap was evaluated under several angles. First, the coverage of SSMap was 'indirectly' assessed in terms of the relative new and missing mappings when compared to the other resources (Table 2). In general, it was found that SSMap provided a considerable number of new mappings as compared to SIFTS and DR PDB (Table 2), and these new mappings were not the simple result of a more recent update of SSMap. The manual verification of a subset of data (200) showed that the quality of new mappings was good. As for missing mappings in SSMap, it was shown that over 90% of these mappings were deliberately removed because of ambiguities and uncertainties in the automatic attribution of the PDB chain to a UniProtKB entry. Taken together, the coverage of SSMap could be considered good. Second, the quality of alternative mappings provided by SSMap was evaluated by manually checking subsets of data. Correct alternative mappings in SSMap were found in about 60% of the cases when compared to all other resources (Table 3). While the result was promising, it was hard to conclude as the datasets were small. However, this verification did point out that SSMap alternative mappings could help identify problematic cases to be checked manually. In fact, we found that nearly all the cases verified manually were worth inspection. Sometimes it was even necessary to read the associated paper to make the correct decision. Finally, we assessed if the boundaries provided by mappings in SSMap were of good quality. It was found that few boundaries differed by more than 5 residues in N- or C-terminal when one compared SSMap with SIFTS, or SSMap with DR PDB (Table 5). For these extreme cases, they were often due to the differences in the methodology as discussed above. Therefore, it appears that, overall, SSMap provides mappings of comparable (if not better) quality when compared to other resources. More importantly, SSMap constitutes a completely independent mapping. In fact, it is worth noting that while we compared SSMap to three resources, they are all more or less related: most of the DR PDB lines were provided by SIFTS, and PDBSWS used information from DR PDB.

As one of the main objectives of this work is to ensure that UniProtKB provides up-to-date and good quality cross-references to PDB as well as to facilitate the annotation of 3D-structure-derived information, it is worthwhile highlighting certain aspects of SSMap that may help this task. First, in terms of the curation of DR PDB lines, SSMap should be able to provide complementary data (Table 2, about 18,472 new mappings corresponding to 7,793 PDB entries) and highlight problematic cases for manual curation. The latter consists of, on the one hand, ambiguous or uncertain mappings deliberately omitted from the final mapping (Table 2, missing mappings) and, on the other hand, the alternative mappings proposed by SSMap. Apart from this, a small number of obsolete PDB chains present in DR PDB (and other resources) were also identified in this study and they should be removed. Second, in terms of annotation of protein features, SSMap provides specific mappings to splice isoforms and inteins (e.g. PDB entry 2DHJ chain A mapped to UniProtKB isoform Q5T5U3-3). While the number of the mappings specific to splice isoforms is still modest (486), this number will certainly increase with the rising interest on isoforms. Currently, the mapping presents a good starting point for possible isoform-specific feature annotation. It can also be used in more global studies which aim to understand the impact of splicing events on protein structures. Besides mappings to alternative sequence forms, SSMap also offers all the alignments between a UniProtKB sequence and PDB structures sharing over 70% sequence identity. This resource is important for the curation process, as well as research aiming to accelerate annotation by deriving rules for automatic features propagation. In a larger scale, the mapping provided by SSMap is currently employed in the internal annotation platform SAALSA (Semi-Automated Annotation from Local Structural Analysis). This front-end to the SSMap database helps annotators to curate DR PDB lines. Moreover, it aids annotators to generate coherent annotation from structural information for a number of features (such as modified residues, metal and ligand binding sites) in UniProtKB/Swiss-Prot entries. Finally, it is important to mention that the update frequency of SSMap ensures that annotators can have access to the most recent structural data.

One of the important observations in this study is that there is no unique solution to the UniProt-PDB mapping problem. Depending on the mapping methods used, the results can differ in a fraction of cases. For SSMap, we noted several points for improvement. First, the manual inspection of 200 new mappings identified 2 cases for which the protein sequences are not yet available in UniProt. This was due to the fact that a certain tolerance in taxonomy matching was allowed in the mapping process. In the future, perhaps only perfect taxonomy matching should be accepted for automatic mapping. Second, while comparing the boundaries of different mappings, it was noted that artifact sequences (cloning artifacts, His-tag) were sometimes included in the alignment. This error could be avoided by improving the algorithm to detect and rectify these cases. Similarly, errors were noted in SIFTS that could potentially be corrected (e.g. errors due to the presence of discontinuous sequences). Indeed, by having a completely independent set of mappings, a more systematic feedback and curation process could now be envisaged with SIFTS. Through this process, the scientific community can benefit from high quality DR PDB lines with minimal errors and inconsistencies.

Since UniProtKB release 12.5, the SSMap UniProt-PDB mapping is used together with SIFTS to produce updates of DR PDB lines. SSMap is available for download from . A 3D visualization tool based on SSMap alignments is also available to help users visualize annotated features in UniProtKB sequences on related 3D-structures with over 70% sequence identity.

## Conclusion

A new UniProt-PDB residue-residue mapping resource SSMap has been created. A systematic comparison with other existent resources showed that SSMap is able to provide a set of independent mappings which complement the current data. The comparison further allows to identify general problems in UniProt-PDB mappings so that both the accuracy and the coverage of the UniProt cross-references to PDB, as well as the optimal use of structural information for feature annotation can be enhanced. SSMap mapping is currently used to provide PDB cross-references in UniProtKB.

## Competing interests

The authors declare that they have no competing interests.

## Authors' contributions

FPAD conceived and implemented the SSMap method and did the comparison work. YYL supervised the study, participated in improving the method and in interpreting the results. YYL and FPAD wrote the manuscript. All authors read and approved the final manuscript.
